# Current Approaches To and Implementation of Information Environment Assessments in the Context of Public Health: Rapid Review

**DOI:** 10.2196/72165

**Published:** 2025-11-06

**Authors:** Becky K White, Fernan Talamayan, Tara Rose Aynsley, Richard Bahizire Riziki, Catherine Bertrand-Ferrandis, Kai Von Harbou, Rocio Lopez Inigo, Thomas Moran, Reuben Samuel, David Scales, Sandra Varaidzo Machiri

**Affiliations:** 1 Department of Country Readiness Strengthening, World Health Organization, Avenue Appia 20, Geneva, 1211, Switzerland, 41 22 791 21 11; 2 Regional Office for South-East Asia, World Health Organization, Delhi, India; 3 Country Office for DRC, World Health Organization, Kinshasa, Congo; 4 Olylo, Paris, France; 5 Africa Infodemic Response Alliance, Regional Office for Africa, World Health Organization, Brazzaville, Congo; 6 Department of Medicine, Weill Cornell Medical College, New York, NY, United States

**Keywords:** information environment, infodemic, assessment, public health, risk communication

## Abstract

**Background:**

With the advances in digital information sharing channels, democratization of content, and access, as well as social shifts in information exchange, we live in increasingly complex information environments. How people process and manage this is layered with multiple determinants that can impact information seeking, health behaviors, and public health. Understanding the dynamics of the information environment in priority populations and its impact on communities and individuals is critical for those working in public health and health emergencies.

**Objective:**

This study aimed to provide an overview of the approaches to and implementation of information environment assessments as they relate to public health and health emergencies.

**Methods:**

We conducted a rapid scoping review of the approaches to, and implementation of information environment assessments. The search followed guidance from the Joanna Briggs Institute on conducting systematic scoping reviews, and our reporting is in line with the PRISMA (Preferred Reporting Items for Systematic Reviews and Meta-Analyses) guidelines for scoping reviews. We included both academic and gray literature in the English language. As this is an emerging field, an additional step involved input from an informal expert group to identify any further tools or approaches. Studies that assessed, described, or discussed approaches to assessing the information environment were included. We excluded papers where the information environment was not the primary focus, or the focus was on individual components only. Two authors (BKW and SVM) independently screened results for inclusion.

**Results:**

A total of 17 publications were identified through the structured literature and internet searches, with an additional 5 sourced from the informal expert group. The review highlighted a significant variety in the breadth and number of domains covered in an assessment, including information needs, seeking, access, production, engagement, information quality, and reach. Some assessments adopted a comprehensive, systems-oriented approach, examining factors influencing information beyond the individual level to encompass broader systemic dynamics, while others were significantly narrower in scope.

**Conclusions:**

The COVID-19 pandemic has intensified interest in understanding how the information environment shapes people’s access to, engagement with, and ability to act on health information. Assessing the information environment is a critical step in identifying and understanding barriers and facilitators that impact different populations and identifying opportunities for strengthening systems. However, a universally accepted approach for such assessments in public health and health emergencies is currently lacking. This paper contributes to the literature by synthesizing current knowledge on assessment tools and frameworks, providing a foundation for future research and development in this area.

## Introduction

With the advances in, and increasing access to digital information sharing channels, democratization of content, and social shifts with high levels of connectivity [1], we live in increasingly complex information environments. Information environments are determinants of health, directly impacting health decision-making and also indirectly affecting health by informing people’s choices about other health determinants [2]. In any given day, a person can be exposed to health information through various social media channels, from family and friends, from colleagues, in their workplace, through advertising, through media, and from health care workers—all delivered, amplified, and shared through different platforms, modes, and volumes. Factors in an information environment may also contribute to reducing an individual’s exposure and access to health information, also impacting their ability to effectively process and manage health information and make informed decisions. The dynamics shaping the information environment are broad and include structural, organizational, community, and individual factors. Navigating this is layered with multiple determinants such as social and commercial determinants of health.

During a health emergency, health information seeking, exchange, and engagement can be heightened, creating an infodemic. This can make it hard for people to find the information they need, in formats they trust, from locally relevant and appropriate sources. An infodemic is the overabundance of information, accurate and otherwise, in the physical and digital space that accompanies an acute health event [3]. The impact of infodemics on public health has been extensively documented [4-7], yet methods to prevent, assess, and identify early opportunities to mitigate the impact are less well-defined. Understanding the information environment in a priority population is important for those working on public health and health emergencies. It can provide valuable insights to guide risk communication, community engagement, and infodemic management activities, as well as a broader range of public health initiatives and system strengthening. The World Health Organization (WHO) framework for “Strengthening the global architecture for health emergency prevention, preparedness, response, and resilience” has community protection as a core pillar [8]. Initiatives that support assessing the Information environment are a key component in informing strategies to promote and support community resilience.

Mapping the multiple factors impacting an individual’s experience in accessing, processing, and engaging with information has been described in several different ways in the literature. There is significant disparity in comprehensiveness between labels and accompanying tools defining the scope. In the literature, the terms “information environment” and “information ecosystem” have been used interchangeably. The Carnegie Endowment for International Peace defines an information environment as “The space where human cognition, technology, and content converge” [9]. Internews, a global media support nonprofit, uses the term information ecosystem and defines it as, “The combination of information providers, channels, platforms and tools that people have at their disposal to access, share and create information. Internews Information Ecosystem Framework maps the available information supply, demand and the complex relationships between the different actors, to make it easier to understand and improve the overall quality of information available in a specific community” [10]. And Radsch [11] states, “A healthy information ecosystem is a well-balanced and diverse system where information is created, shared, and used responsibly.”

In a public health context, the WHO has defined an information environment as “The entire universe of information that individuals, communities, health systems, and societies inhabit. This constitutes structures, agents, and systems that influence information seeking, access, sharing, and use. In an infodemic management context, the information environment is overlayed with the considerations of the quality of the health information content and sources, because this affects how the information environment affects people’s adherence to health guidance and health behaviors” [12]. This is different from an information ecosystem that is posited to be “more specific to a community that has norms and structures for how information is shared, reacted to and acted on” [12]. For the purposes of this research, we use this definition of information environment, noting that there is not established consensus on these definitions among scholars. Table 1 summarizes the differences between the 2 terms as per this definition. This paper seeks to understand the different approaches to information environment assessments and the domains that are included. It is intended to provide an overview of the current literature, identify strengths and weaknesses in current approaches, and be used to inform the development of comprehensive assessments.

**Table 1.** Comparison of terms, information environment, and information ecosystem.

Term	Information environment	Information ecosystem
Definition	The entire universe of information individuals, communities, health systems, and societies inhabit.	More specific to a particular community and its norms and structures for how information is shared, reacted to, and acted on.
Scope	Broader; it includes structures, agents, systems, and processes.	More focused; centers on a specific community and how the community engages with information.
Key components	Structures, agents, and systems influencing information seeking, access, sharing, and use.	Norms, rules, and established practices within a community that govern information sharing and responses.
Application	Applies to large-scale settings (eg, societies, health systems) with an emphasis on access, engagement, and use of information.	Applies within specific communities, emphasizing norms and practices of that group.
Infodemic management context	Concerned with quality and accessibility of health information and how these influence health-related behaviors and adherence to public health guidance.	More relevant to community-level behaviors and practices concerning health information.
Influence on behavior	Affects society-wide adherence to public health guidance and behaviors.	Shapes community-specific responses to shared information.
Example	Global or national health information systems, media landscapes, social media platforms, and their interaction with public health.	Local health organizations, community groups, and small closed social networks (eg, WhatsApp or Viber groups) with established norms.
Assessment	Comprehensive, aiming to evaluate the strength of systems that influence information production, dissemination, and use.	Focused on how specific communities react to, use, and share information.

## Methods

### Overview

We conducted a rapid scoping review of the approaches to, and implementation of, information environment assessments as they relate to public health. We followed guidance from the Joanna Briggs Institute on conducting systematic scoping reviews [13] and our reporting is in line with the PRISMA (Preferred Reporting Items for Systematic Reviews and Meta-Analyses) guidelines for scoping reviews [14] (Checklist 1). We included both academic and gray literature. As this is an emerging field, an additional step involving input from an informal expert group to identify any further tools or approaches is needed. Experts were invited to participate in the informal group due to their current activities in, and knowledge of, the information environment space.

### Review Objective

This study aimed to provide an overview of the approaches to, and implementation of information environment assessments as they relate to public health.

### Review Questions

We aimed to seek answers to the following research questions. First, what research questions and phenomena of interest do researchers and practitioners intend to address when assessing information environments? Second, what approaches to defining and assessing information environment have been outlined and implemented? And finally, what domains are covered and how are they assessed?

### Search Strategy

The search strategy included both academic and gray literature. To source unpublished literature, including government reports and news articles, the Google search engine was used. Search terms were adapted, and the first 100 results were scanned in each search. The search strategy for academic literature was developed in line with MEDLINE and then adapted for other databases. We initially trialed the search with MEDLINE, testing for keywords. Other databases included in the search include ACM Digital Library, EBSCO, and Embase. The search was carried out in June 2024.

### Search Terms

As our rapid review was focused on understanding the different definitions and assessments associated with information environments, our search terms were developed to focus on the core concepts of the information environment (“information ecosystem” OR “information environment” OR “information landscape”) and the assessment component (assessment OR mapping OR analysis OR tool OR scan). These were initially tested in MEDLINE and revised with the initial searching results. We limited search results to the past 10 years (2014-2025). An adapted search was used for Google.

### Inclusion and Exclusion Criteria

We included studies, reports, or documents that assess, describe, or discuss approaches to assessing the information environment in the English language. There was no geographical restriction. As the aim was to map the existing tools and approaches across published and unpublished literature, we included reports, perspective and discussion papers, and government and NGO documents, including reports and presentations, as well as academic publications.

We excluded papers where the discussion of assessing an information environment was not the primary focus or where it contained insufficient relevant content. We excluded any papers that referred only to individual components of the information environment, for example, those looking just at algorithms, social media, or image analysis. While we did include papers reporting on information environment assessments outside of the domain of health (such as military, communication, and so on), those hyperfocused on specific and narrow circumstances, that is, the corporate setting of an educational facility, or where the “information landscape” was defined only as an audit of digital products, were excluded. For the Google search, we were looking for reports or publications by organizations or professional bodies that described approaches to assessing the information environment.

### Study Selection

The selection of articles was defined by the inclusion and exclusion criteria. In line with the iterative nature of scoping reviews, these criteria were reviewed as the search progressed. The Rayyan platform was used for deduplication and screening. Two investigators (BKW and SVM) independently screened article titles and abstracts, excluding irrelevant studies. The full text was reviewed for articles deemed possibly relevant, or where inclusion was difficult to ascertain from the title and abstract. Any discrepancies were resolved via discussion.

In the Google search, where multiple returns pointed to the same organizational approach or methodology (eg, there were several Internews reports returned that followed similar methods), we combined them into 1 entry.

### Expert Input

A virtual meeting was held with an informal group of experts providing an opportunity for identification of any further tools and assessments. These experts were primarily practitioners working in the information environment space and were from a diverse range of countries and organizations. Experts were invited to participate in the informal group due to their current activities in, and knowledge of, the information environment space. Publications were provided to the research team and included after the literature assessment where there was a publicly available version.

### Summarizing and Reporting

The data charting method was trialed with the first 3 papers and revised. Data were collected regarding study characteristics, area of focus and publication intent, domains covered and data collection for assessment method.

## Results

### Overview

**Figure 1.**
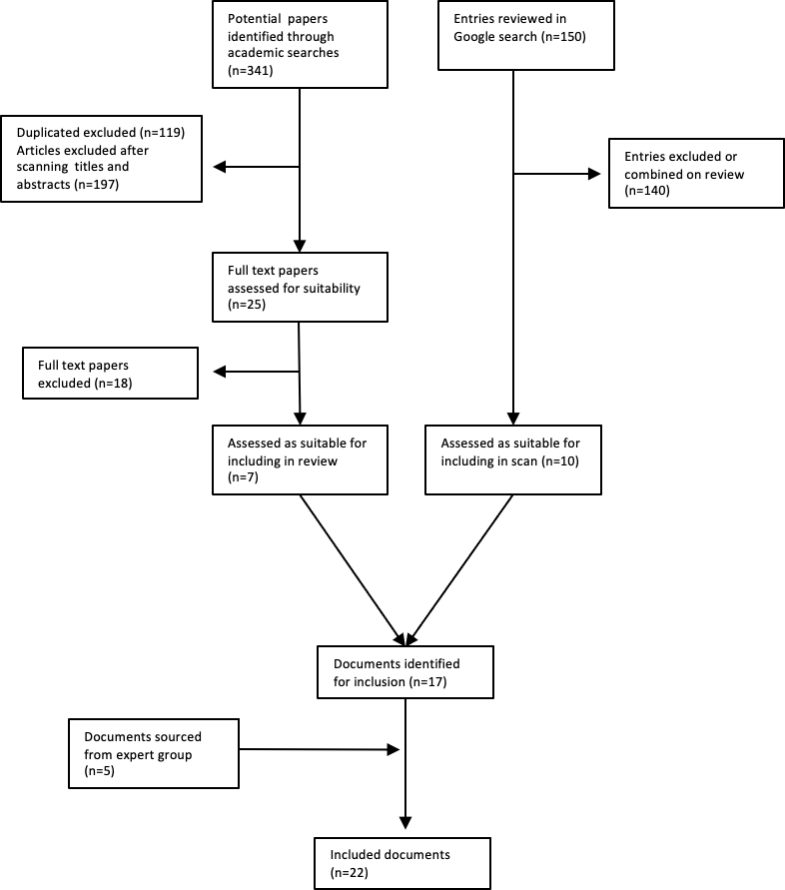
Publication selection process.

A total of 17 publications were identified for inclusion through the structured literature and online searches, and an additional 5 were identified through the expert group. Figure 1 illustrates the study selection process. While we did exclude publications that were overly narrow in their scope (eg, only including social media), we did include those that used a single data collection point (ie, interviews) to measure multiple domains. The selected papers focused on health and emergencies, as well as other areas, such as education, defense, and corporate settings. Where we identified multiple reports using the same methodology from an organization, we combined them into one entry. An example of this is Internews [15], which conducted and published assessments in several geographical settings, as well as the CDAC (Communicating with Disaster Affected Communities) Network [16]. Table 2 describes the characteristics of the included publications, and findings are summarized here. The use of “information ecosystem” or “information environment” in this table reflects the preference of the paper’s authors, while the definitions used in the core paper are as explained earlier.

**Table 2.** Characteristics of included publications.

Information	Intent or aim	Focus area	Domains	Data use	Notes on approach
Ye and Yang [17] (conference paper)	To provide theoretical guidance for the governance of university network public opinion.	University network public opinion information ecosystem.	Information (quality, type, quantity, security, authenticity, and timeliness), information subject (communication ability, attitude tendency, user situation, and platform function), and information environment (technical, cultural, physical, economic, and institutional and science environment).	—^a^	Proposes analytic hierarchy process to combine qualitative and quantitative data.
Savoia et al [18] (journal article)	To articulate a conceptual framework in support of evaluation activities in emergency risk communications.	Emergency risk communications evaluation framework.	Information environment level: timeliness, news coverage, message content (literacy, framing, transparency, self-efficacy, consistency, and rumors).	—	Proposes information environment as a component of emergency risk communication framework.
Moldovan-Johnson et al [19] (journal article)	To describe and assess how patients with cancer navigate the information environment with regard to their illness.	Information environment of patients with cancer.	Active information seeking, exchange, and engagement with different data sources including digital and community. Media and health care workers.	Literature review, expert consultation, repeated surveys with patients.	Information environment assessment focuses on information seeking and engagement.
Ramírez et al [20] (journal article)	To identify and critically evaluate public sources of information about the causes and controllability of air pollution and its health effects, and potential disparities in information reach and utility.	Environmental health.	Information sources: public, expert, and media; information quality: breadth, depth, and type; and information reach: vulnerable groups, access to technology, and language.	Analysis of existing communicators and communication strategies, interviews with stakeholders, and community residents.	Focus on environmental risk communication. Findings show themes around information quality, sources, and reach.
Estrada et al [21] (journal article)	To describe a participatory multilevel, ecological information inequity intervention in a rural, majority-Latino community.	Rural, Latino community in the United States.	Informational (where health information can be provided), conversational (where residents feel comfortable discussing health issues), and connection (where a relationship exists).	Communication asset mapping: Workshops and interviews with organizations and residents.	Describes a participatory health communication asset mapping research process tailored for rural community.
El Tímpano [22] (NGO^b^ report)	To learn what issues are most important to Latino immigrants and what gaps and opportunities exist in connecting them to the news and information they need to be informed and engaged.	Migrant communities.	Structural: health, housing, and safety. Information: access, language, and understandability.	Desktop research, surveys, community conversations, outreach, and immersion.	Community-based assessment conducted by people with significant ties to the local community.
Mazumdar et al [23] (journal article)	To highlight the interconnectedness of different actors in the agricultural communities and the complexities involved in establishing trust of information.	Rural agricultural communities.	Information needs, sources, processes, social context, and knowledge. Skills and knowledge. Use of technology.	Field visits, interviews, focus groups.	The paper includes exploration of different social contexts and power dynamics in the information landscape in rural areas.
Internews [15] (NGO report)	To identify how information flows through communities.	Humanitarian settings, emergency response, and vulnerable communities.	Information needs, information landscape, production and movement of information, dynamic of access, use of information, impact of information, social trust, and influencers in the community.	Interviews with health system stakeholders, media organizations, population and humanitarian organizations.	Broad assessment model across 7 domains. Applied to different settings (including as Listening Post Collective in the United States) such as Columbia, Thailand, and California.
Wanless and Shapiro [9] (report)	To define and understand the conditions within the information environment that can foster democratic societies and encourage active citizen participation.	Global.	Human cognition, technology, and content. Information processing, social norms, access, and platforms.	Social media, political data, education levels, access to technology, press freedom, perception and trust data, media consumption data, and policy and legal data.	The paper proposes how the CERN (European Center for Nuclear Research) model can be used for studying the information environment.
Radsch [11] (network collective report)	To map the multidimensional aspects of an information ecosystem that can be used at various levels of analysis.	Global.	ICT^c^ infrastructure (platforms, penetration, resource allocation, commercial impacts, and content moderation), dynamic conditions (access and connectivity, data and digitalization, media system, new media, transparency and accountability, and anchor institutes and keystone species).	—	Proposes model with multiple domains across different levels of analysis.
GAO^d^ [24] (government report)	To better inform officials on the use and protection of the information environment including effects on government department mission, threat actors, threat actions, institutional challenges, and emerging technologies.	US National security.	Cognitive dimension: beliefs, norms, vulnerabilities, motivations, emotions, experiences, morals, education, mental health, identities, and ideologies. Physical dimension: human beings, command and control facilities, newspapers, books, communication towers, computer servers, laptops, smart phones, and tablets. Informational dimension: collection, processing, storage, dissemination, and protection of information.	Questionnaires to defense organizations, interviews, and desktop review.	Identifies 3 broad dimensions of the information environment – cognitive (human-centric), informational (data-centric), and physical (tangible and real-world).
RAND [25] (research institute report)	To harness the power of information to increase efficiencies, improve security, and deny access and advantages to adversaries.	US National security.	Information operations; social media; data analytics and big data; intelligence collection and analysis; and information security, privacy, and information-sharing.	—	Bibliography of operational reports in the information environment. Most focused on Dept of defense efforts.
Röttger and Vedres [26] (academic report)	To describe and understand research on the information environment and effects on individuals and groups.	Research.	Exposure characterizes (encounters between individuals and information content). Engagement (interaction between the individual and the information they are exposed to). Digital technologies (including information generation, seeking, and sharing).	—	Interdisciplinary literature review identifies various components.
NATO^e^ Strategic Communications Centre of Excellence [27] (simulation platform)	To meet training needs of defense sector for understanding the information environment.	US National security.	Information environment infrastructure (networks), dynamics (processes), and audiences (behavior, opinions, and characteristics).	Simulated platform for training purposes.	Aims to provide dynamic training opportunities through an AI-generated simulation platform across the information environment.
CDAC Network [16] (NGO report)	To provide responders with the evidence needed to adapt and expand their communication, community engagement, and accountability efforts.	Humanitarian settings.	Information needs, media landscape, preferred channels, information sources and verification mechanisms, information access, trust, population, and worker perspectives.	Document review, focus groups, key informant interviews.	Assessment methodology applied to different settings such as Syria and Türkiye.
UNHCR^f^ [28] (report)	To identify the information and communication needs of refugees and migrants, as well as the most appropriate channels for information sharing.	Refugees and migrants.	Information needs, channels used, barriers to access, and reliable information sources.	Semistructured interviews.	Assessment focused on information needs and barriers and facilitators in a transit center in North Macedonia.
Canadian Digital Media Research Network [29] (technical report)	To enhance collective understanding of the stable and dynamic dimensions of the Canadian information ecosystem, articulate its vulnerabilities, and characterize current and emerging information threats.	As the Canadian information environment relates to politics, media, and state of democracy.	Vulnerabilities: inequity, toxicity, polarization and insularity, and trust. Threats: misinformation and foreign influence. Engagement: trends in news appetite and online engagement with politicians.	Digital trace data (social media), opinion tracking (monthly surveys), media monitoring.	Monthly assessment reports, first on May 2024.
Fleisher [30] (book chapter)	To describe an environmental scanning process to support corporate strategic decision-making.	Corporate settings.	Institutions: including policy, government, regulatory agencies, and legislation. Stakeholders: key actors that can influence policy. Arenas: (platforms, media, and elections). Issues: (gaps or controversy between stakeholders and actions or behaviors). Processes: (legal processes, judicial bodies).	—	Information on setting up an organizational focused process.
Bridgman et al [31] (book)	To describe the relationship between digital media and democracy by investigating elements of the Canadian information ecosystem.	Canada digital ecosystem.	Digital media focus. Concentration of influence and fragmentation.	Social media data and survey data	Discussion on Canadian information environments and assessments by “Media ecosystem observatory” as part of Canadian Digital Media Research Network.
Ahmad [32] (report)	To identify the various information ecosystems in place among the youths in Malaysia.	Malaysian youth.	Media consumption, media ownership, community access, information and media sustainability, opportunities and needs assessment.	Survey and focus groups.	Assessment and methods by Center for Independent Journalism, supported by Internews.
USAID^g^ [33] (toolkits and reports)	To describe an assessment process for the digital ecosystem.	Global.	Framework includes digital economy (trade, e-commerce, financial services, talent pool, and startups), digital infrastructure and adoption (connectivity, security literacy, divide, and affordability), digital society, rights and governance (rights, governance, government, civil society, and media), and cross-cutting (inclusion, cybersecurity, emerging technology, and geopolitical positioning).	Desk research, interviews.	Includes assessment toolkit, factsheets, framework, templates, and country assessment examples.
IPIE^h^ [34] (report)	To understand how technology experts perceive the varied features of, and threats to, the information environment in their countries of expertise.	Global.	Top 5 features of healthy information environments: availability of accurate information, diversity, literacy, technical infrastructure, and accountability. Information threat actors: politicians, social media platforms, and governments.	Survey of scientists.	Global survey on information environment components conducted in English, Arabic, Chinese, French, and Spanish.

a Not applicable.

b NGO: nongovernment organization.

c ICT: information and communication technologies.

d GAO: Government Accountability Office.

e NATO: North Atlantic Treaty Organization.

f UNHCR: United Nations High Commissioner for Refugees.

g USAID: United States Agency for International Development.

h IPIE: International Panel on the Information Environment.

### Focus Area and Population Groups

The focus of the publications differed from universally applicable models or approaches, sometimes repeated in different settings, to those developed for, or conducted with, specific communities. Of the total, 3 of the publications focused on national security, with all 3 having a United States focus [24,25,27]. Of those that described assessments designed with specific communities or settings in mind, focus areas included a university network [17], patients with cancer [19], corporate settings [30], environmental health [20], a rural Latino community in the United States [21], a migrant community in the United States [22], rural agricultural communities in Bangladesh [23], Malaysian youth [32], and the Canadian digital ecosystem [29,31]. International organizations described a focus on humanitarian settings and emergency response in a range of countries such as Colombia, Syria, North Macedonia, and Türkiye [15,16,28,33].

### Domains Covered

There was a wide approach to the breadth and number of domains covered. Some publications focused on information needs, seeking, access, production, and engagement [18,19,21,22,26,28], while others included indicators on information quality and reach [17,20]. Some publications focused primarily on media, including media consumption and ownership, community engagement, and access [16,32], while others were focused on the digital environment only [31,33]. Most described domains in a neutral manner; however, 2 publications specifically sought to identify “threat actors” or “threats” to the information environment [16,34]. Several publications discussed frameworks or approaches to an information environment assessment that were comprehensive and broad, including factors that look beyond the individual focus to take a systems approach to influencing factors. These included the model proposed by Radsch [11] that emphasized the interconnectedness of different institutions, systems, and sectors. Another example is Fleisher [30] who considered wider arenas, such as elections and media, as well as legal and judicial processes. Trust as a specific domain was evident in 3 publications [15,16,29].

### Operational Publications and Tools

Publications included in the review were a mix of proposed models with indicators, research projects, and operational tools. In total, 12 publications described assessments that had been completed. Both Internews and CDAC Network have multiple assessments using similar methods that have been used in several different settings globally. For Internews, this includes Colombia, Thailand, and California [15], while the CDAC network had conducted assessments in countries such as Syria and Türkiye [16]. In 2024, a new technical report was introduced in Canada, issuing monthly Information Ecosystem Situation reports [29]. The United States Agency for International Development Digital Ecosystem Country Assessment has accompanying documents such as a toolkit, templates, framework, and research guide [33]. Most operational tools included a combination of desk research, digital indicators, and community or stakeholder input in the form of interviews or a survey.

## Discussion

### Principal Findings

This paper outlines the current landscape of both academic and gray literature focused on approaches to assessing and understanding the information environments in which we live. Since 2020, the importance of the information environment in public health has grown significantly with the COVID-19 pandemic underscoring how a poor information environment can hinder people’s access to accurate, reliable, and appropriate information. In response, a wide range of methods, tools, research, theory, and practices have emerged [35]. Understanding the dynamics of an information environment is vital in identifying barriers and facilitators and helping to inform action. This could include informing risk communication, community engagement, and infodemic management interventions, as well as providing guidance for policy, health service access, health system strengthening, and health literacy interventions, among others. With this in mind, and to acknowledge the delay that can occur in publishing academic literature, we structured this review to look beyond published papers and incorporate reports, methods, tools, and documents sourced through an internet Google search, and through the input of experts working in the field.

We found significant variation in the domains considered to constitute the information environment and how they were assessed, as well as variation in terminology and how they were described. This presented challenges in comparisons between approaches. Many papers looked at information production, but only a few included consideration of exposure, amplification structures, and consumption. The intention and breadth of scope in each example were different, which guided the individual approaches. Some focused on media environments only and did not include wider health system determinants or consider them only in an emergency context and not in routine work. Few considered the breadth of domains included in the WHO definition of an information environment, such as the structures, agents, and systems that influence information seeking, access, sharing, and use. The model described by Radsch (2022) [11] considered multidimensional aspects across a broad number of domains, such as commercial impacts, platforms, and policy, but there was no description of how this could be implemented. Community-level assessments reported benefit in building on community networks, strengths, and existing relationships [21-23].

While the literature has been maturing, health and humanitarian emergencies, including the COVID-19 pandemic, have highlighted the need for practical, implementable solutions to understand and assess infodemics and information environments for those working on the ground [36,37]. Some of the most comprehensive models in this review were those that had been developed by international nongovernment organizations (NGOs) and deployed in the field. Internews has conducted a number of information environment assessments across a range of settings, including supporting other organizations in their work [15]. Their work has been evolving over time in humanitarian and emergency situations. The CDAC Network has also applied its method to assessments in countries such as Syria and Türkiye [16]. The United States Agency for International Development Digital Ecosystem Country Assessment model included factors such as cybersecurity, geopolitical positioning, and affordability, alongside literacy and media [33]. Interestingly, trust was mentioned as a specific domain in 3 documents we assessed, all of them global operational documents [15,16,29].

How we as individuals navigate our information environment can change daily and is influenced by a range of factors, including the health of ourselves and our families, and the attitudes of a health care worker delivering the information. It is also influenced by things out of an individual’s control, such as the availability of information in relevant languages, market factors, trust in institutions and community, commercial and social determinants of health, the regulatory environment, and the media landscape. While individual social determinants of health, such as access to education and social and neighborhood environments, can impact how individuals navigate the information environment, the broad-ranging impact has led to calls for information environments to be included as an element in social determinants of health models [2].

It has been noted that assessing the information environment is incredibly complex [3]. While we can ask about and ascertain the media that people are exposed to, developing ways to measure the engagement with it over time, the change in consumption and individual processing patterns driven by emotional state, the interaction between media consumption and offline interactions, and conversations remain very challenging. In a similar vein, identifying and defining health system policy, commercial influences, and ethical practices is one thing, but understanding their impact on each other, and on the information environment as a whole, is another. Here, scholars and practitioners can face challenges similar to those who are studying social network analysis in terms of defining and measuring ties and impacts between and within factors over time [38]. As an information environment or ecosystem can change rapidly, particularly in emergency situations, another challenge for assessment tools is likely to be how changes to, and impacts both on and within environments and communities can be measured over time while remaining flexible enough to incorporate changes and the evolution of factors. Here, there may be opportunities to build on systems thinking models that seek to understand interrelationships between complex components in relation to a common purpose [39].

The publications reviewed for inclusion in this paper revealed a gap between the literature and the tools and documents being produced for use in the field and by international organizations to operationalize work rapidly. This could be due to the publishing delay, with time taken for papers to be processed through the peer-review process, but it could also suggest a wider divergence between research and practice. This has been reflected in another recent paper, with the authors noting the diversity of factors needing consideration in an information environment and issuing an urgent call for information scientists to design and guide the development of systems [37]. As these processes and frameworks continue to mature, more targeted collaborative research and partnerships between academia and practitioners are needed to ensure approaches employed on the ground are evidence-based, inform best practice, and that practitioners are supported to document their work and assess relevance and transferability across settings. There is a need for robust models that are comprehensive and based on the best global evidence and practice, as well as the need for them to remain agile to respond to the ever-changing conditions and factors impacting an information environment. In addition, more evidence is needed to understand how we can translate research on these complex interactions into a workable assessment that can be used by national and subnational organizations to inform their work in a way that is rapid, cost-effective, and comprehensive.

### Strengths and Limitations

Due to the rapid nature of the review, decisions were made to narrow the scope, which introduced limitations. The first of these is the inclusion of English-language articles only. This limited the breadth of the findings, and it would be valuable to include languages other than English in future research. Although limited to the English language only, the papers included here cover different geographical and cultural contexts.

As our rapid review was focused on understanding the different definitions and assessments associated with information environments, our search terms were confined to those specifically related to reporting on information environments. This narrowed the findings. The terminology in this space is not consistent, and there have been precursor terms. For example, in political and communication science, some researchers have used “media landscape” or “media ecosystem” as broadcast media was the primary contributor to what we now call an information environment. Our search limited findings by not exploring the different ways assessments such as this may have been defined and described previously (eg, “media ecosystems”). In addition, considering the broad nature of the work, many of the findings focused on external factors, rather than individual processing and theory of human interactions with the information environment. The inclusion of assessments beyond the realm of those specifically focused on health was a strength that enabled a broader view.

### Conclusion

The COVID-19 pandemic has intensified interest in understanding how the information environment shapes people’s access to, engagement with, and ability to act on health information. Assessing the information environment is a critical step in identifying and understanding barriers and facilitators that impact different populations. However, a universally accepted approach for such assessment is currently lacking. This paper contributes to the literature by synthesizing current assessment tools and frameworks, providing a foundation for future research and development in this area. It aimed to answer questions about approaches to, and the intent of, information environment assessments and the domains covered. Findings showed significant variation, including in the domains considered to constitute the information environment. The reported gaps between research and practice reveal a need for more collaborative projects. Increasing understanding of what constitutes a healthy information environment and the factors that impact this, in both a positive and negative way, can help to ensure individuals and communities are best placed to receive, understand, and act on good accurate health information, to better protect public health, build trust, and improve health outcomes. As the impact of information environments on public health outcomes continues to grow, there is a need for comprehensive tools that can be deployed in a rapid manner, with practical applicability.

## Supplementary material

10.2196/72165 Checklist 1 PRISMA ScR checklist.

## Abbreviations

CDAC: Communicating with Disaster Affected Communities
NGO: nongovernment organization
PRISMA: Preferred Reporting Items for Systematic Reviews and Meta-Analyses
WHO: World Health Organization
